# Combination of Serum Amyloid A and C-Reactive Protein Exhibit Synergistic Effect in Angiogenesis by Inducing Inflammation and Vascular Network

**DOI:** 10.3389/fonc.2020.576207

**Published:** 2020-12-08

**Authors:** Dan Liu, Yonghe Chen, Yunxiu Wang, Mangjuan Lei, Lin Chen, Rongliang Liang, Zhaomin Cheng, Wen Shi, Huimin Wang, Li Lin, Lina Wang, Fujia Lin, Haibiao Lin, Wanli Liu

**Affiliations:** ^1^ Department of Laboratory Medicine, Guangdong Provincial Hospital of Chinese Medicine, Guangzhou, China; ^2^ Department of Clinical Laboratory, Sun Yat-sen University Cancer Center, State Key Laboratory of Oncology in South China, Collaborative Innovation Center for Cancer Medicine, Guangzhou, China; ^3^ Department of Gastrointestinal Surgery, The Sixth Affiliated Hospital, Sun Yat-sen University, Guangzhou, China

**Keywords:** binding of C-reactive protein and serum amyloid A, inflammation, vascular network, lung cancer, promotion

## Abstract

The role of angiogenesis in tumor progression has been recognized as one of the hallmarks of cancer, but the mechanism of its action remains unclear. Inflammatory markers serum amyloid A (SAA) and C-reactive protein (CRP) are proposed to play causal roles in the development of various disorders, including malignancies. Previously, we identified the complex of CRP and SAA (CRP-SAA) with diagnostic and prognostic value better than either one of them in the serum of lung cancer patients. In this study, we further explored the stimulation function of CRP-SAA on angiogenesis and inflammation. To explore possible mechanisms, microarray datasets were downloaded from the Gene Expression Omnibus (GEO) database and multi-bioinformatics analysis revealed that THP-1 and human umbilical vein endothelial cells (HUVECs) responded to SAA stimulation with upregulation of two pro-angiogenic cytokines in common, i.e., C-X-C motif ligand 6 (*CXCL6*) and *CXCL8*, which were validated by subsequent experiments *in vitro*. CRP had weak effects as a single stimulus, but it can efficiently potentiate the SAA induction of cytokines, which was stronger than the sum of the both (*P* < 0.001). The synergistical effect of the combination of CRP and SAA enhanced HUVECs transwell and constricted morphology by upregulating the pro-angiogenic genes. These results indicated that the binding of CRP and SAA acted synergistically in pro-angiogenesis by increasing inflammation and inducing vascular network.

**GRAPHICAL ABSTRACT:**
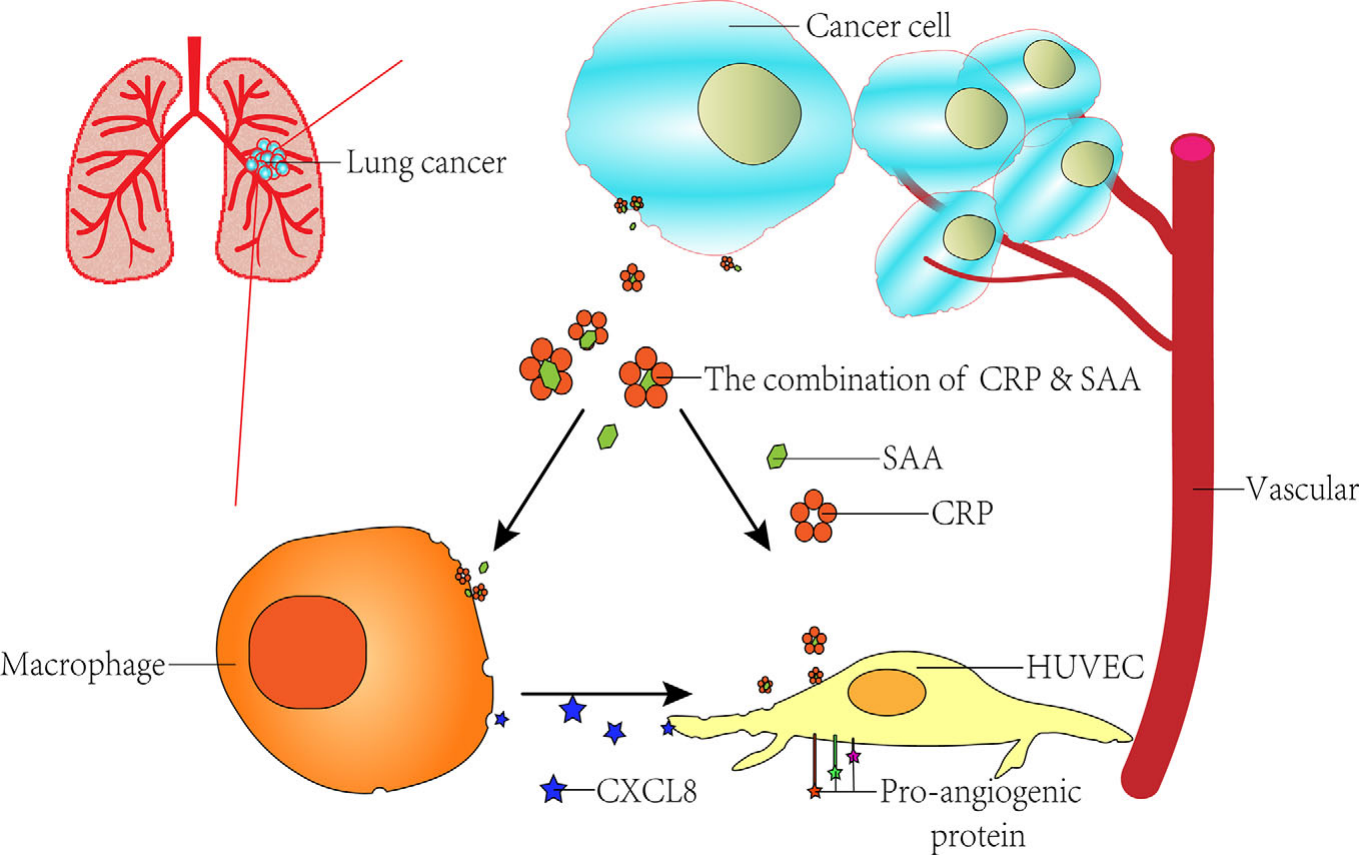


## Introduction

Lung cancer (LC) is the most common cause of cancer-associated death worldwide. In the last decades, angiogenesis has aroused strong clinical interest (1). Anti-angiogenic therapy has been widely administered in several types of human cancers, but is still limited by the challenges of cytotoxicity and reduced efficiency (1). New anti-angiogenic drugs that may help prevent (“angio-prevention”) or treat advanced tumor stages by inducing tumor regression or inhibiting tumor progression have been clinically investigated (2).

Various factors and cellular mechanisms have been described as responsible for the initiation of blood vessel formation in tumors (3). The angiogenic switch can be triggered by genetic changes in tumor cells, or by recruitment of immune cells in tumor-associated inflammation (4). Inflammation is long thought to involve the progress of vascular epithelial cells response and proliferation, which make it as important as angiogenesis (5).

Serum amyloid A (SAA) is an acute phase protein with multiple immune functions, including inducing the synthesis of relating cytokines and being chemotactic for neutrophils and immune cells (6). In a large number of phenomenological studies of alterations in cancer serum proteins from normal controls, levels of SAA were found elevated in a relatively early stage (7). SAA has been evaluated as a possible serum biomarker for many tumors including ovarian (8), lung (9), renal (10–12), colorectal (13), etc (14, 15). Further evidence linking SAA to tumor behavior has been investigated in promoting metastasis (16), angiogenesis, and inflammation of inducing macrophages to the M2 type (17).

The classic members of the pentraxin family have a high affinity for many types of autologous and external ligands, and C-reactive protein (CRP) is a typical one (18). During the process of tumor formation, the inflammatory cytokines produced by the tumor microenvironment stimulate hepatocytes, leading to the elevated level of serum CRP (19). CRP can enter the tumor microenvironment through circulation and interact with various autologous and external ligands by binding as complex, which can in turn play a key role in activating or inhibiting tumor-associated macrophages (20). Besides working in combination with various proteins, the elevated CRP alone is also a strong indicator of multiple types of cancers (21), including LC (22).

In our previous study, proteomics analysis specifically identified CRP-SAA complexes from serum samples of lung cancer patients, which could be used as diagnostic and independent prognostic markers for early-stage lung cancer patients (23). In this study, we further explored the promoting role CRP-SAA played in angiogenesis by activating tumor-inflammatory responses and inducing vascular network.

## Method

### Microarray Data

In the discovery step, we identified datasets for comparing mRNA expression with or without SAA treatment on THP-1 and HUVEC. Gene expression profiles of GSE28785 (THP-1) and GSE6241 (HUVEC) were obtained from the National Center for Biotechnology Information (NCBI) GEO database (https://www.ncbi.nlm.nih.gov/geo/) (24). GSE6241 was based on the GPL570 platform, while GSE28785 was based on the GPL6947 platform. The overlap in the upregulated gene sets were analyzed with a Venn plot by Funrich. The expression difference between the control and stimulated cells were present by volcano plots and heatmaps performed by SangerBox. A protein–protein interaction (PPI) network was established by the STRING database, and hub genes were visualized by Cytoscape (25).

### Cell Culture

Human monocytic leukemia cell line, THP-1, was obtained from the Cell Bank of the Type Culture Collection of the Chinese Academy of Sciences. The cells were cultivated at 37°C in a 5% CO_2_ incubator at a density of 5 × 10^5^ cells/mL in RPMI 1640 medium supplemented with 10% fetal bovine serum (FBS) and 1% penicillin/streptomycin solution (Biological Industries, Israel). In all experiments, THP-1 monocytes transformed into adherent macrophages by culturing in six-well plates treated with 100 nM phorbol 12-myristate 13-acetate (PMA) for 24 h.

Primary human umbilical vein endothelial cells (HUVECs, Clonetics) were cultivated in EGM-2MV medium (Clonetics) and 5% FBS. Cells were then trypsinized and reseeded until the cells reached 90% confluence. HUVECs from passages 3–5 were used in all of the following experiments.

### In Vitro Stimulation and Cytokines Evaluation

In CRP and SAA treatment studies, THP-1 or HUVEC cells were cultured to the control, i.e., 0.1% bovine serum albumin (BSA) in phosphate buffer saline (PBS), or CRP (Abcam, 1 μg/mL) or SAA (SantaCruz, 0–1 μg/mL) or both of them for 2–6 h. At the end of the incubation, cell pellets were collected for RNA isolation while the supernatants were for the quantification of chemokines by ELISA.

### RNA Extraction and qPCR

Total RNA was extracted using the Trizol reagent (Invitrogen, USA) according to the manufacturer’s instruction. Reverse transcription of total RNA (1 μg) was performed using SuperScript II reverse transcriptase. The quantification of C-X-C motif chemokine ligand 6 (*CXCL6*), *CXCL8*, vascular endothelial growth factor (*VEGFA*), intercellular cell adhesion molecule-1 (*ICAM1*), vascular cell adhesion molecule-1 (*VCAM1*), E-selectin (*SELE*), and reference gene (β-actin) were performed in triplicate on a LightCycler^®^ 480 II (Roche, Applied Science) using a SYBR green-based assay (BioRad, USA). The primers used in the qPCR reaction are shown in **Table 1**.

**Table 1:** Primers used for the polymerase chain reaction.

Target gene	Sequence
*CXCL6*	F: 5′- GTAGCCTCCCTGAAGAACGG-3′ R: 5′- GGTCCAGGGATCTCCAGAAA-3′
*CXCL8*	F: 5′- AATGAAAAGATGAGGGTGCAT-3′ R: 5′- GCTTGTGTGCTCTGCTGTCT-3′
*VEGFA*	F: 5′-CTACCTCCACCATGCCAAGT-3′ R: 5′-CCATGAACTTCACCACTTCGT-3′
*ICAM1*	F: 5′-GAGGAAGGAGCAAGACTCAA-3′ R: 5′-AGCATACCCAATAGGCAGCAAG-3′
*VCAM1*	F: 5′-CCTGCCATTGGAATGATAA-3′ R: 5′-TGCTTCTACAAGACTATATGAC-3′
*SELE*	F: 5′-GTTTGGTGAGGTGTGCTCATT-3′ R: 5′-CATTTTACCACTTGGCAGGAA-3′
*β-actin*	F: 5′-CGCGAGAAGATGACCCAGAT-3′ R: 5′-GGGCATACCCCTCGTAGATG -3′

### Measurement of Chemokines Released From Macrophages

The concentration of chemokines CXCL6 and CXCL8 in the supernatant were assayed using ELISA kits (eBioscience). The procedures for the assays were in accordance to the product manual.

### Transwell Migration Assays

Transwell assays were performed with 8 μm pore size Transwell chambers (BD Bioscience). Upper surfaces of the Transwell inserts were coated with 50 μl Matrigel and incubated for 1 h at 37°C for gelling. HUVECs (2 × 10^4^ cells/well) were suspended in 250 μl of serum-free RPMI-1640 medium with CRP (1 μg/mL) or SAA (1 μg/mL) or both of them in the upper chamber, and 500 μl complete M199 medium was added to the lower chamber. After 18 h incubation at 37°C, non-invasive cells on the upper membrane surfaces were removed. The Transwell chambers were fixed with 4% paraformaldehyde and stained hematoxylin. Cell invasion was quantified by counting cells on the lower surface using a microscope at ×100 magnification. The results were the means calculated from three independent replicates of each experiment.

### Cell Morphology Analysis

Matrigel was thawed at 4°C overnight, then 150 μl of Matrigel was coated on each well of 48-well plates and incubated at 37°C for 1 h. HUVECs (5 × 10^4^ cells) were added in 500 μl complete M199 medium with stimulation at atmosphere of 37°C and 5% CO_2_ for 18 h. The cell surface area was analyzed using ImageJ software, and for each condition a minimum of 150 cells from three experiments were examined.

## Results

### SAA Can Stimulate THP-1 and HUVEC by Up-Regulating *CXCL6*, *CXCL8*, and Pro-Angiogenic Genes in Early Stage

Over the past few decades, bioinformatics analyses have been widely used to explore genetic variation, which helps us identify differentially expressed genes (DEG) and functional pathways related to cancerogenesis and progression (26). We downloaded the global gene expression profiles of THP-1-derived macrophages and HUVECs stimulated by SAA from the GEO database under accession numbers GSE28785 (THP-1) and GSE6241 (HUVEC).

**Figure 1:**
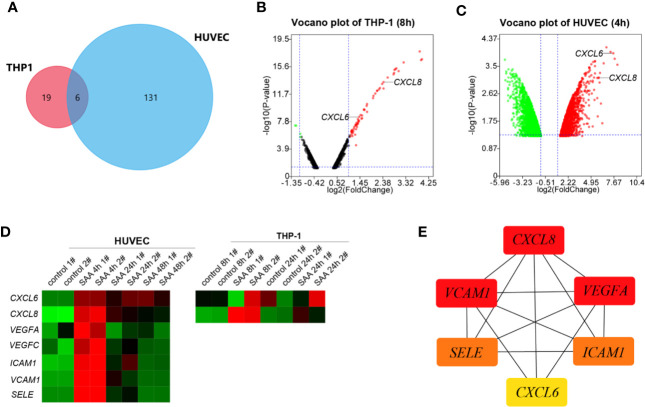
**(A)** Venn plot for the six overlapped genes identified from GSE28785 and GSE6241. **(B)** Volcano plots of SAA treatment on global gene expression in THP-1 macrophages for 8 h from GSE28785. **(C)** Volcano plots of SAA treatment on global gene expression in HUVEC for 4 h from GSE6241. **(D)** Heatmap was drawn to describe levels of potential hub gene expressions. **(E)** The six genes were ranked by the CytoHubba plugin. The lines show the interactions between them. The red and yellow nodes represent the rank of hub genes from high to low according to the degree. The more forward ranking is represented by a redder color.

A Venn-diagram-based analysis framework showed six genes, including tumor necrosis factor, alpha-induced protein 6 (*TNFAIP6*), *CXCL8*, serpin family b member 2 (*SERPINB2*), tumor necrosis factor, alpha-induced protein 2 (*TNFAIP2*), and *CXCL6* were upregulated both in stimulated THP-1 (8 h) and HUVEC (4 h), which suggested that the genes were closely associated with activation and communication of the two types of cells stimulated by SAA (**Figure 1A**).

*CXCL6* and *CXCL8* were among top ten up-regulated pathways and genes in the early stages (8 h) (27) of activation of THP-1 (**Figure 1B**). The HUVEC also increased *CXCL6* and *CXCL8* with high fold change in 4 h of stimulation (**Figure 1C**). However, there was a noticeably reduced level of the up-regulated gene in the sustained stimulation for 24 h and 48 h (**Figure 1D**).

Besides *CXCL6* and *CXCL8*, pro-angiogentic genes (*VEGFA*, *VEGFC*, *ICAM1*, *VCAM1*, and *SELE*) also responded to SAA with similar trends (**Figure 1D**). CytoHubba in Cytoscape 3.8.0 identified networks from complex interactome. All of six hub genes were upregulated, and the top three hub genes were sequentially as follows: *CXCL8*, *ICAM1*, and *VEGFA* (**Figure 1E**).

### The CXCL8 and CXCL6 Were Induced by SAA in a Concentration-Dependent Way in THP-1

The circulating concentration of SAA in healthy individuals has a wide range with an average value of 10 μg/mL (28). However, since the vascular development occurs in the arterial walls where less SAA is found, a lower concentration of 0.25–1 μg/mL SAA was used (27). The THP-1 cells and supernatant were collected at 2 h, 4 h, and 6h of SAA stimulation. The mRNA and proteins of CXCL6 and CXCL8 were detected by qPCR and ELISA, respectively.

**Figure 2:**
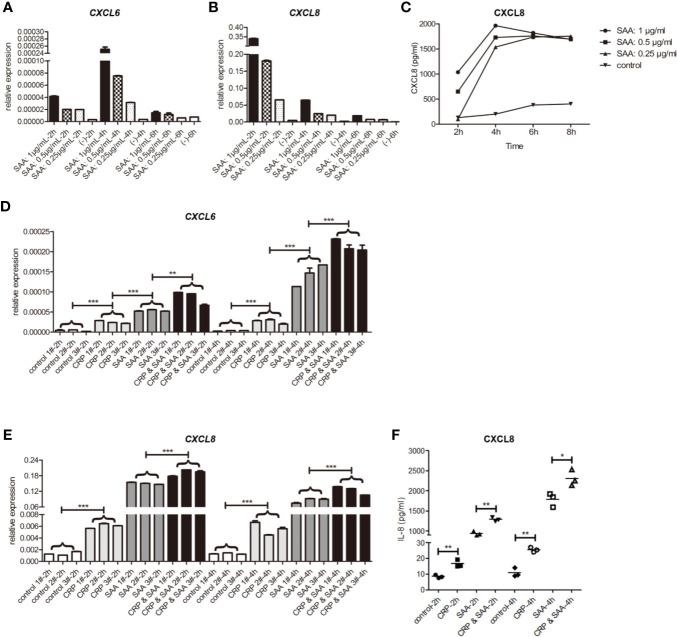
The relative expression of mRNA of CXCL6 **(A)** and CXCL8 **(B)** stimulated by SAA of different concentrations (0, 0.25, 0.5, 1μg/mL) and durations (2 h, 4 h, 6 h) in THP1. **(C)** The levels of CXCL8 in the supernatant were detected by ELISA. The relative expression of mRNA of CXCL6 **(D)** and CXCL8 **(E)** in THP1 stimulated by the control, CRP (1μg/mL), SAA (1μg/mL), or CRP (1μg/mL) and SAA (1μg/mL) for different duration (2 h, 4 h) from three independent replicates of each experiment. **(F)** The levels of CXCL8 in the supernatant were detected by ELISA. The asterisk indicates significant difference in cell surface area compared to the control, using the Student t-test (*P < 0.05, **P < 0.01, ***P < 0.001).

Similar to the previous studies (27), the peak of *CXCL6* mRNA was at the early stage of 4 h stimulation (**Figure 2A**). In addition, we found the peak of *CXCL8* mRNA was as early as 2 h (**Figure 2B**), and the levels of relative expression of *CXCL8* were much higher than *CXCL6*. Both of them were SAA concentration-dependent.

In the subsequent ELISA tests of cell supernatant, we detected high levels of CXCL8 dependent on SAA concentration. They reached a peak at 4 h and declined slightly afterward. The concentration of CXCL6 was too low to be detected (**Figure 2C**).

Overall, we hypothesized that the CXCL8 responded to SAA stimulation with more sensitivity and higher fold change than CXCL6, and the protein was secreted into the supernatant soon after by THP-1.

### The Combination of CRP-SAA Further Increased *CXCL6* and CXCL8 in THP-1

To detect the role of the combination of SAA and CRP, we added only CRP or SAA or both of them in THP-1 in three independent parallel experiments. The statistical analysis revealed that CRP had light but significant activation of *CXCL6* (**Figure 2D**) and CXCL8 (**Figures 2E, F**) on THP-1. Moreover, the combination of CRP and SAA can obviously improve the expression of *CXCL6* (**Figure 2D**) and CXCL8 (**Figures 2E, F**) more than sum of them. We hypothesized that CRP can potentiate the inflammatory effect of SAA on THP-1.

### The Combination of CRP-SAA Increased the Migration and Morphology Disruption of HUVEC by Induction of Angiogenesis-Associated Genes

**Figure 3:**
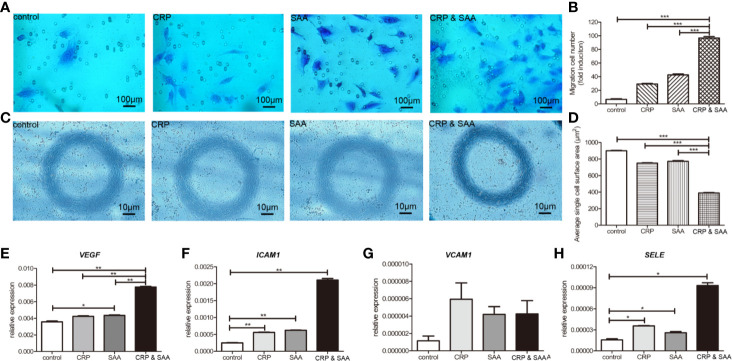
Migration of HUVECs cultured in medium with or without stimulation was performed in a Transwell chamber with 8.0 μm pore size. Representative images of HUVECs that migrated through the membrane **(A)** and the number of cells that migrated through the membrane/field **(B)** were presented. Values were expressed as the mean ± SD. *n* = 3. **(C)**. HUVECs were stimulated for 18 h and the representative images were captured to show the cell shape of HUVECs. The combination of CRP and SAA produced cells with a constricted morphology with long membrane extensions compared to negative control or either one of them. **(D)** Quantification of the change in cell morphology by measurement of cell surface area. For each condition, a minimum of 150 cells from three experiments was examined. The relative expression of angiopoietin-like proteins, including VEGF **(E)**, ICAM1 **(F)**, VCAM1 **(G)**, and SELE **(H)** in the groups of HUVEC treated with the control, or CRP, or SAA, or combination of CRP and SAA. The asterisk indicates significant difference in cell surface area compared to the control, using the Student t-test (*P < 0.05, **P < 0.01, ***P < 0.001).

Since the migration and invasion of endothelial cells through basement membranes was a key step in the development of new blood vessels, we assessed the effect of CRP-SAA on endothelial cell HUVEC migration. The HUVEC exhibited promoted migration after sole stimulation against the negative control, but the combination of CRP and SAA enhanced a more severe migration with a synergistic effect (**Figures 3A, B**).

The combination of CRP and SAA induced endothelial cells with a more constricted cell morphology characterized by exaggerated membrane extensions than single CRP or SAA, whereas the normal control endothelial cells displayed a normal cell shape (**Figures 3C, D**).

Because these endothelial cells significantly altered migration ability and cell morphology, we collected the cells for further evaluation. **Figures 3E–H** indicated that the levels of pro-angiogenic factors, including *VEGFA*, *ICAM1*, and *SELE*, significantly increased in CRP- and SAA-treated medium compared with the control or single stimulation. The *VCAM1* was upregulated without significance, probably due to the small sample size. These results indicated that simultaneously elevated CRP and SAA triggered the angiogenic switch by induction of angiogenic factors in HUVEC.

**Figure 4:**
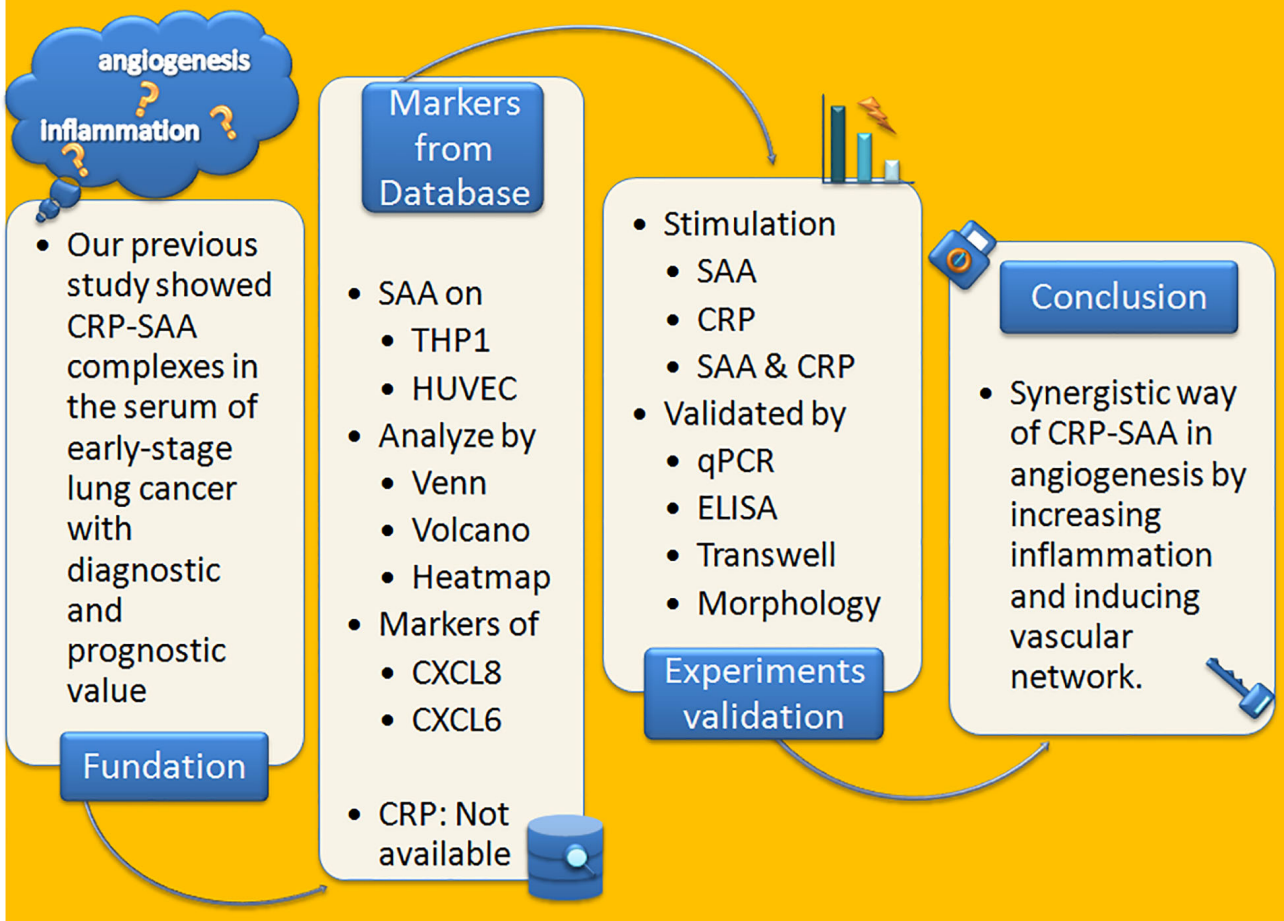
A flowchart of the project steps.

The **Figure 4** summarized a flowchart of the project steps.

## Discussion

In our previous study, proteomic analysis specifically identified the complex of CRP-SAA from serum samples of lung cancer patients with a significant difference from the normal candidates. The elevation of CRP-SAA was associated with lower survival rates for lung cancer patients, which made it a better prognostic marker than SAA or CRP, especially in early-stage patients (23). In this study, we further explored the promotion role of CRP-SAA in angiogenesis and inflammation.

Tumor angiogenesis occurs through several different biological processes, orchestrated by a series of secreted factors and signal transduction pathways, and may involve the participation of non-endothelial cells (such as immune cells) (29). Microvessels and immune cells are the main components of the tumor microenvironment (TME). The crosstalk between cancer cells and TME, and among TME cells sustains tumor growth, invasion, and metastasis (30).

Previously, local SAA production within cancer cells introduced SAA as a tumor marker for its linkage with tumor metastasis, inflammation, and angiogenesis (17). CRP, a member of the pentraxin family, was found as an important prognostic marker in patients with several malignancies, including lung (31), urological (32), pancreatic (33), hepatocellular (34), and colorectal cancers (35). The CXCL6 and CXCL8, in addition to the initially identified proinflammatory function, are known for their ability to behave in a disparate manner in the regulation of angiogenesis (4, 36–38). In cancer, an elevated level of CXCL8 was proved to be associated with worse survival of melanoma (39).

In this study, two mRNA microarray datasets from GEO were downloaded and analyzed to obtain DEGs between SAA-stimulated THP-1/HUVEC cells and the control. Both *CXCL6* and *CXCL8* were identified to be upregulated with “high-confidence,” i.e., high fold change and small *P*-value, by multi-bioinformatic approaches, especially in the early stage.

Along with *CXCL6* and *CXCL8*, *VEGF*, *ICAM1*, *VCAM1*, and *SELE* can also be directly induced by SAA in heatmap analysis, which was consistent with previously substantial evidence in dermal human microvascular endothelial cells (HMVECs) (40), rheumatoid arthritis (RA) synovial fibroblasts (41), and giant cell arteritis (GCA) myofibroblasts (42). But in our study, the subsequent construction of the hub gene network helped to catch sight of the most potentially functional *CXCL8*, *VEGFA*, and *VCAM1* among the six hub genes closely associated with SAA-induced angiogenesis.

In the co-cultivation experiments of THP-1, we further shortened the stimulation time to 2 h, and found the *CXCL8* responded to SAA with the highest fold change in 2 h by qPCR studies. ELISA was performed to evaluate a high level of CXCL8 protein reached to peak in 2 hours subsequently. But the *CXCL6* did not change as highly and sensitively as *CXCL8*, and the concentration of CXCL6 protein was too low to be detected. The induction was in a SAA-concentration dependent manner. Similar to the results of bioinformatic analysis, these detections *in vitro* also confirmed the importance of CXCL8 in SAA-induced THP-1 inflammatory activation and communication.

Previous studies revealed that CRP can increase the expression of *CXCL8* (19, 43, 44), *VEGF (* 45), and adhesion molecules, including *ICAM1* and *VCAM1 (* 46–48). In our study, CRP alone could stimulate *CXCL6* and CXCL8 in THP-1, and *VEGF*, *ICAM1*, and *SELE* in HUVECs slightly but significantly in the early stage of stimulation. When the CRP was added in combination with SAA, CRP might efficiently potentiate the effect of cytokine induction, especially CXCL8. We hypothesized the CRP acted synergistically in the SAA inflammatory promotion in THP-1.

In terms of the angiogenesis, the effect of the combination of CRP and SAA on HUVECs transwell and constricted morphology was stronger than the sum of both inductions. Further, we demonstrated that the synergistic effect on these changes may be related to the enhancement in angiogenesis stimulators, including *VEGF*, *ICAM1*, and *SELE*.

Previous studies have shown that some angiogenic factors may also be involved in inflammation and worsen clinical outcomes. VEGF, ICAM1, and VCAM1 were found to suppress tumor immunity by inhibiting the maturation of dendritic cells, and induce immunosuppressive cells such as regulatory T cells, tumor-associated macrophages, and myeloid-derived suppressor cells (49, 50). CXCL8 signaling could not only promote angiogenesis, and proliferation and survival of endothelial cells, but also increase progression of cancer cells, infiltrating neutrophils with both pro- and antitumor properties (51) at the tumor site (52). The complex of CRP-SAA could increase the CXCL cytokines and angiopoietin-like proteins in the synergistic way, so we hypothesized the CRP-SAA participated in a wide range of processes that contributed to tumor development and progression.

In conclusion, the preceding may be the mechanisms of the association between elevation of CRP-SAA and lung cancer poor prognosis. Our study may highlight new potential targets for the development of future therapeutic strategies.

## Data Availability Statement

The datasets presented in this study can be found in online repositories. The names of the repository/repositories and accession number(s) can be found in the article/supplementary material.

## Author Contributions

DL: wrote the article. YC: wrote the article. YW: software. ML: statistics. WS: supervision. HW: supervision. RL: supervision, LL, LW: statistics, FL: software. WL: supervision. HL: supervision. All authors contributed to the article and approved the submitted version.

## Funding

This work was supported by Science and Technology Program of Guangzhou, China (201704020176), Doctoral Initiating Foundation of Guangdong for Natural Sciences (2018A030310507), China Postdoctoral Science Foundation (2018M633038) and the National Natural Science Foundation of China (81271902).

## Conflict of Interest

The authors declare that the research was conducted in the absence of any commercial or financial relationships that could be construed as a potential conflict of interest.

## References

[1] Abdalla AME Xiao L Ullah MW Yu M Ouyang C Yang G Current Challenges of Cancer Anti-angiogenic Therapy and the Promise of Nanotherapeutics. Theranostics (2018) 8(2):533–48. 10.7150/thno.21674 PMC5743565 29290825

[2] Carmeliet P Jain RK Principles and mechanisms of vessel normalization for cancer and other angiogenic diseases. Nat Rev Drug Discov (2011) 10(6):417–27. 10.1038/nrd3455 21629292

[3] Hicklin DJ Ellis LM Role of the vascular endothelial growth factor pathway in tumor growth and angiogenesis. J Clin Oncol (2005) 23(5):1011–27. 10.1200/JCO.2005.06.081 15585754

[4] Lugano R Ramachandran M Dimberg A Tumor angiogenesis: causes, consequences, challenges and opportunities. Cell Mol Life Sci (2020) 77(9):1745–70. 10.1007/s00018-019-03351-7 PMC7190605 31690961

[5] Imhof BA Aurrand-Lions M Angiogenesis and inflammation face off. Nat Med (2006) 12(2):171–2. 10.1038/nm0206-171 16462798

[6] Eklund KK Niemi K Kovanen PT Immune functions of serum amyloid A. Crit Rev Immunol (2012) 32(4):335–48. 10.1615/CritRevImmunol.v32.i4.40 23237509

[7] Rosenthal CJ Sullivan LM Serum amyloid A to monitor cancer dissemination. Ann Internal Med (1979) 91(3):383–90. 10.7326/0003-4819-91-3-383 289303

[8] Edgell T Martin-Roussety G Barker G Autelitano DJ Allen D Grant P Phase II biomarker trial of a multimarker diagnostic for ovarian cancer. J Cancer Res Clin Oncol (2010) 136(7):1079–88. 10.1007/s00432-009-0755-5 PMC2874491 20082099

[9] Sung HJ Ahn JM Yoon YH Rhim TY Park CS Park JY Identification and validation of SAA as a potential lung cancer biomarker and its involvement in metastatic pathogenesis of lung cancer. J Proteome Res (2011) 10(3):1383–95. 10.1021/pr101154j 21141971

[10] Tolson J Bogumil R Brunst E Beck H Elsner R Humeny A Serum protein profiling by SELDI mass spectrometry: detection of multiple variants of serum amyloid alpha in renal cancer patients. Lab Invest (2004) 84(7):845–56. 10.1038/labinvest.3700097 15107802

[11] Paret C Schön Z Szponar A Kovacs G Inflammatory protein serum amyloid A1 marks a subset of conventional renal cell carcinomas with fatal outcome. Eur Urol (2010) 57(5):859–66. 10.1016/j.eururo.2009.08.014 19747761

[12] Bozinovski S Vlahos R Anthony D McQualter J Anderson G Irving L COPD and squamous cell lung cancer: aberrant inflammation and immunity is the common link. Br J Pharmacol (2016) 173(4):635–48. 10.1111/bph.13198 PMC4742298 26013585

[13] Himbert C Ose J Lin T Warby CA Gigic B Steindorf K Inflammation- and angiogenesis-related biomarkers are correlated with cancer-related fatigue in colorectal cancer patients: Results from the ColoCare Study. Eur J Cancer Care (2019) 28(4):e13055. 10.1111/ecc.13055 PMC6639140 31016796

[14] Zhou J Sheng J Fan Y Zhu X Tao Q He Y Association between serum amyloid A levels and cancers: a systematic review and meta-analysis. Postgraduate Med J (2018) 94(1115):499–507. 10.1136/postgradmedj-2018-136004 30341230

[15] Sack GH Jr. Serum amyloid A - a review. Mol Med (Cambridge Mass) (2018) 24(1):46. 10.1186/s10020-018-0047-0 30165816 PMC6117975

[16] Hansen MT Forst B Cremers N Quagliata L Ambartsumian N Grum-Schwensen B A link between inflammation and metastasis: serum amyloid A1 and A3 induce metastasis, and are targets of metastasis-inducing S100A4. Oncogene (2015) 34(4):424–35. 10.1038/onc.2013.568 24469032

[17] Knebel FH Uno M Galatro TF Bellé LP Oba-Shinjo SM Marie SKN Serum amyloid A1 is upregulated in human glioblastoma. J Neurooncol (2017) 132(3):383–91. 10.1007/s11060-017-2386-z 28283801

[18] Pathak A Agrawal A Evolution of C-Reactive Protein. Front Immunol (2019) 10:943. 10.3389/fimmu.2019.00943 31114584 PMC6503050

[19] Sproston NR Ashworth JJ Role of C-Reactive Protein at Sites of Inflammation and Infection. Front Immunol (2018) 9:754. 10.3389/fimmu.2018.00754 29706967 PMC5908901

[20] Pilling D Galvis-Carvajal E Karhadkar TR Cox N Gomer RH Monocyte differentiation and macrophage priming are regulated differentially by pentraxins and their ligands. BMC Immunol (2017) 18(1):30. 10.1186/s12865-017-0214-z 28619036 PMC5472910

[21] Dolan RD Laird BJA Horgan PG McMillan DC The prognostic value of the systemic inflammatory response in randomised clinical trials in cancer: A systematic review. Crit Rev Oncol Hematol (2018) 132:130–7. 10.1016/j.critrevonc.2018.09.016 30447918

[22] Iivanainen S Ahvonen J Knuuttila A Tiainen S Koivunen JP Elevated CRP levels indicate poor progression-free and overall survival on cancer patients treated with PD-1 inhibitors. ESMO Open (2019) 4(4):e000531. 10.1136/esmoopen-2019-000531 31555483 PMC6735669

[23] Zhang XY Zhang G Jiang Y Liu D Li MZ Zhong Q The prognostic value of serum C-reactive protein-bound serum amyloid A in early-stage lung cancer. Chin J Cancer (2015) 34(8):335–49. 10.1186/s40880-015-0039-1 PMC4593389 26264146

[24] NCBI Resource Coordinators. Database resources of the National Center for Biotechnology Information. Nucleic Acids Res (2016) 44(D1):D7–19. 10.1093/nar/gkv1290 26615191 PMC4702911

[25] Doncheva NT Morris JH Gorodkin J Jensen LJ Cytoscape StringApp: Network Analysis and Visualization of Proteomics Data. J Proteome Res (2019) 18(2):623–32. 10.1021/acs.jproteome.8b00702 PMC6800166 30450911

[26] Qian W Xiaoyi W Zi Y Screening and Bioinformatics Analysis of IgA Nephropathy Gene Based on GEO Databases. BioMed Res Int (2019) 2019:8794013. 10.1155/2019/8794013 31392215 PMC6662497

[27] Leow KY Goh WW Heng CK Effect of serum amyloid A1 treatment on global gene expression in THP-1-derived macrophages. Inflamm Res (2012) 61(4):391–8. 10.1007/s00011-011-0424-4 22228103

[28] Lappalainen T Kolehmainen M Schwab U Pulkkinen L Laaksonen DE Rauramaa R Serum concentrations and expressions of serum amyloid A and leptin in adipose tissue are interrelated: the Genobin Study. Eur J Endocrinol (2008) 158(3):333–41. 10.1530/EJE-07-0598 18299466

[29] Viallard C Larrivée B Tumor angiogenesis and vascular normalization: alternative therapeutic targets. Angiogenesis (2017) 20(4):409–26. 10.1007/s10456-017-9562-9 28660302

[30] Nazemi M Rainero E Cross-Talk Between the Tumor Microenvironment, Extracellular Matrix, and Cell Metabolism in Cancer. Front Oncol (2020) 10:239. 10.3389/fonc.2020.00239 32175281 PMC7054479

[31] Yang JR Xu JY Chen GC Yu N Yang J Zeng DX Post-diagnostic C-reactive protein and albumin predict survival in Chinese patients with non-small cell lung cancer: a prospective cohort study. Sci Rep (2019) 9(1):8143. 10.1038/s41598-019-44653-x 31148582 PMC6544765

[32] Zhou L Cai X Liu Q Jian ZY Li H Wang KJ Prognostic Role of C-Reactive Protein In Urological Cancers: A Meta-Analysis. Sci Rep (2015) 5:12733. 10.1038/srep12733 26235332 PMC4522672

[33] Szkandera J Stotz M Absenger G Stojakovic T Samonigg H Kornprat P Validation of C-reactive protein levels as a prognostic indicator for survival in a large cohort of pancreatic cancer patients. Br J Cancer (2014) 110(1):183–8. 10.1038/bjc.2013.701 PMC3887299 24201751

[34] Zheng Z Zhou L Gao S Yang Z Yao J Zheng S Prognostic role of C-reactive protein in hepatocellular carcinoma: a systematic review and meta-analysis. Int J Med Sci (2013) 10(6):653–64. 10.7150/ijms.6050 PMC3619114 23569429

[35] Kwon KA Kim SH Oh SY Lee S Han JY Kim KH Clinical significance of preoperative serum vascular endothelial growth factor, interleukin-6, and C-reactive protein level in colorectal cancer. BMC Cancer (2010) 10:203. 10.1186/1471-2407-10-203 20465852 PMC2886042

[36] Heidemann J Ogawa H Dwinell MB Rafiee P Maaser C Gockel HR Angiogenic effects of interleukin 8 (CXCL8) in human intestinal microvascular endothelial cells are mediated by CXCR2. J Biol Chem (2003) 278(10):8508–15. 10.1074/jbc.M208231200 12496258

[37] Keane MP Belperio JA Xue YY Burdick MD Strieter RM Depletion of CXCR2 inhibits tumor growth and angiogenesis in a murine model of lung cancer. J Immunol (2004) 172(5):2853–60. 10.4049/jimmunol.172.5.2853 14978086

[38] Kitadai Y Haruma K Mukaida N Ohmoto Y Matsutani N Yasui W Regulation of disease-progression genes in human gastric carcinoma cells by interleukin 8. Clin Cancer Res (2000) 6(7):2735–40. 10914718

[39] Jamal R Lapointe R Cocolakis E Thébault P Kazemi S Friedmann JE Peripheral and local predictive immune signatures identified in a phase II trial of ipilimumab with carboplatin/paclitaxel in unresectable stage III or stage IV melanoma. J Immunother Cancer (2017) 5(1):83. 10.1186/s40425-017-0290-x 29157311 PMC5696743

[40] Mullan RH McCormick J Connolly M Bresnihan B Veale DJ Fearon U A role for the high-density lipoprotein receptor SR-B1 in synovial inflammation via serum amyloid-A. Am J Pathol (2010) 176(4):1999–2008. 10.2353/ajpath.2010.090014 20304957 PMC2843487

[41] Mullan RH Bresnihan B Golden-Mason L Markham T O’Hara R FitzGerald O Acute-phase serum amyloid A stimulation of angiogenesis, leukocyte recruitment, and matrix degradation in rheumatoid arthritis through an NF-kappaB-dependent signal transduction pathway. Arthritis Rheum (2006) 54(1):105–14. 10.1002/art.21518 16385502

[42] O’Neill L Rooney P Molloy D Connolly M McCormick J McCarthy G Regulation of Inflammation and Angiogenesis in Giant Cell Arteritis by Acute-Phase Serum Amyloid A. Arthritis Rheum (2015) 67(9):2447–56. 10.1002/art.39217 26016600

[43] Pasceri V Cheng JS Willerson JT Yeh ET Modulation of C-reactive protein-mediated monocyte chemoattractant protein-1 induction in human endothelial cells by anti-atherosclerosis drugs. Circulation (2001) 103(21):2531–4. 10.1161/01.CIR.103.21.2531 11382718

[44] Kibayashi E Urakaze M Kobashi C Kishida M Takata M Sato A Inhibitory effect of pitavastatin (NK-104) on the C-reactive-protein-induced interleukin-8 production in human aortic endothelial cells. Clin Sci (Lond) (2005) 108(6):515–21. 10.1042/CS20040315 15701058

[45] Bello G Cailotto F Hanriot D Kolopp-Sarda MN Latger-Cannard V Hess K C-reactive protein (CRP) increases VEGF-A expression in monocytic cells via a PI3-kinase and ERK 1/2 signaling dependent pathway. Atherosclerosis (2008) 200(2):286–93. 10.1016/j.atherosclerosis.2007.12.046 18280482

[46] Fricke I Mirza N Dupont J Lockhart C Jackson A Lee JH Vascular endothelial growth factor-trap overcomes defects in dendritic cell differentiation but does not improve antigen-specific immune responses. Clin Cancer Res (2007) 13(16):4840–8. 10.1158/1078-0432.CCR-07-0409 17699863

[47] Ohm JE Gabrilovich D II Sempowski GD Kisseleva E Parman KS Nadaf S VEGF inhibits T-cell development and may contribute to tumor-induced immune suppression. Blood (2003) 101(12):4878–86. 10.1182/blood-2002-07-1956 12586633

[48] Tamura R Tanaka T Ohara K Miyake K Morimoto Y Yamamoto Y Persistent restoration to the immunosupportive tumor microenvironment in glioblastoma by bevacizumab. Cancer Sci (2019) 110(2):499–508. 10.1111/cas.13889 30467920 PMC6361613

[49] Harjunpää H Llort Asens M Guenther C Fagerholm SC Cell Adhesion Molecules and Their Roles and Regulation in the Immune and Tumor Microenvironment. Front Immunol (2019) 10:1078. 10.3389/fimmu.2019.01078 31231358 PMC6558418

[50] Carbone C Piro G Merz V Simionato F Santoro R Zecchetto C Angiopoietin-Like Proteins in Angiogenesis, Inflammation and Cancer. Int J Mol Sci (2018) 19(2). 10.3390/ijms19020431 PMC5855653 29389861

[51] Sagiv JY Michaeli J Assi S Mishalian I Kisos H Levy L Phenotypic diversity and plasticity in circulating neutrophil subpopulations in cancer. Cell Rep (2015) 10(4):562–73. 10.1016/j.celrep.2014.12.039 25620698

[52] Waugh DJ Wilson C The interleukin-8 pathway in cancer. Clin Cancer Res (2008) 14(21):6735–41. 10.1158/1078-0432.CCR-07-4843 18980965

